# Improvement of *n-*butanol tolerance in *Escherichia coli* by membrane-targeted tilapia metallothionein

**DOI:** 10.1186/1754-6834-6-130

**Published:** 2013-09-11

**Authors:** Wei-Chih Chin, Kuo-Hsing Lin, Jui-Jen Chang, Chieh-Chen Huang

**Affiliations:** 1 Department of Life Sciences, National Chung Hsing University, Taichung, Taiwan; 2 Vaccine Research and Development Center, National Institute of Infectious Disease and Vaccinology, NHRI, Miaoli, Taiwan; 3 Department of Medical Research, China Medical University Hospital, Taichung 402, Taiwan; 4 Biodiversity Research Center, Academia Sinica, Taipei 11529, Taiwan; 5 Agricultural Biotechnology Center, National Chung Hsing University, Taichung 402, Taiwan

**Keywords:** Metallothionein, n-butanol, OmpC, Tilapia, *E. coli*, Oxidative stress

## Abstract

**Background:**

Though *n-*butanol has been proposed as a potential transportation biofuel, its toxicity often causes oxidative stress in the host microorganism and is considered one of the bottlenecks preventing its efficient mass production.

**Results:**

To relieve the oxidative stress in the host cell, metallothioneins (MTs), which are known as scavengers for reactive oxygen species (ROS), were engineered in *E. coli* hosts for both cytosolic and outer-membrane-targeted (osmoregulatory membrane protein OmpC fused) expression. Metallothioneins from human (HMT), mouse (MMT), and tilapia fish (TMT) were tested. The host strain expressing membrane-targeted TMT showed the greatest ability to reduce oxidative stresses induced by *n-*butanol, ethanol, furfural, hydroxymethylfurfural, and nickel. The same strain also allowed for an increased growth rate of recombinant *E. coli* under n-butanol stress. Further experiments indicated that the TMT-fused OmpC protein could not only function in ROS scavenging but also regulate either glycine betaine (GB) or glucose uptake via osmosis, and the dual functional fusion protein could contribute in an enhancement of the host microorganism’s growth rate.

**Conclusions:**

The abilities of scavenging intracellular or extracellular ROS by these engineering *E. coli* were examined, and TMT show the best ability among three MTs. Additionally, the membrane-targeted fusion protein, OmpC-TMT, improved host tolerance up to 1.5% n-butanol above that of TMT which is only 1%. These results presented indicate potential novel approaches for engineering stress tolerant microorganism strains.

## Background

*n-*Butanol has many advantages over ethanol, including a higher energy density due to two extra carbons, and can be used in gasoline engines without modification. *n*-Butanol is less hygroscopic and evaporative than ethanol and has been recently regarded as a more viable transportation biofuel than ethanol [1]. Additionally, *n-*butanol is also a permitted artificial flavoring and is used in a wide range of industries, including the food and plastic industries [2]. *n*-Butanol often occurs as a metabolic product of the microbial fermentation using sugars and other carbohydrates as carbon sources. However, during the production of *n-*butanol, its accumulation is known to be highly toxic to both natural producers and engineered hosts [3,4]. This toxicity makes it difficult to produce large titers of *n*-butanol at levels needed for economic efficiency.

The cellular membrane is a vital factor that allows for cells to acclimate to external stresses and is also one of the components highly affected by organic solvents [5,6]. Most toxicity researchers have proposed that the plasma membrane is the most affected target of organic solvents and plays a significant role in adapting to stress. Additionally, the length of the carbon backbone of organic solvents could alter the toxicity mechanism; increasing the hydrophobicity of the solvent could also raise the level of toxicity [7]. The long- and short-chain alcohols are known to cause stress during biofuel production by changing membrane fluidity. Ethanol and n-butanol are known to respectively decrease and increase the membrane fluidity [6,8,9]. Understanding the membrane stress response to solvents and alcohols could facilitate engineer-ing microorganisms for improved toxin tolerance. As such, stress responses of organisms such as *E. coli*, to ethanol exposure has been widely studied [10], and information from these studies have been successfully adapted to engineering improved ethanologenic hosts [11]. To understand the effect of *n-*butanol toxicity on the host, cell-wide studies have been conducted to obtain a global view of the *n-*butanol stress-response in transcript, protein, and metabolite levels. In *Clostridium acetobutylicum*, transcript analysis indicated that the primary response was an accumulation of transcripts encoding chaperones, proteases, and other heat shock-related proteins [12]. In *E. coli*, several transcriptional analyses have been performed to understand the stress caused by alcohols including ethanol, *n-*butanol, and isobutanol [13-16]. Additionally, observations from fluorescent dye-staining indicated a large increase in reactive oxygen species during *n-*butanol stress [15]. This increasing oxidative stress is a response of the cell to extracellular xenobiotics, which may mediate macromolecular damage. These free radicals could directly attack the membrane by lipid peroxidation [17].

ROS include molecules that are either oxidants (such as hydrogen peroxide, H_2_O_2_) or reductants (such as the superoxide anion, O_2_^˙−^). All are typical side products of cellular aerobic metabolism. To decrease ROS-generated oxidative damage, microorganisms synthesize many antioxidant enzymes, including catalases, superoxide dismutases and glutathione peroxidase [18,19]. Recently, metallothioneins (MTs), a beneficial antioxidant enzyme that widely occurs in mammals, plants and fungi, has been identified [20]. MTs are heat-stable, low-molecular-weight and cysteine-rich intracellular proteins that are responsible for maintaining the homeostasis of essential metals, such as Cu^2+^, Zn^2+^ and for the detoxification of toxic metal ions, such as Cd^2+^ and Hg^2+^[20-22]. In addition, MTs also play a role as a defense system against oxidative stress through their ROS-targeted scavenging abilities [23]. For example, the tilapia fish (*Oreochromis mossambicus*), which serves as a biomarker for the contamination level of aqueous environments, has the ability to survive in a highly polluted environment because of its MTs function [24,25]. Furthermore, purified tilapia MT (TMT) has been shown to have a higher ability than glutathione (GSH) to scavenge both 2-diphenyil-1-picrylhydrazyl (DPPH^●^) and 2,2-azinobis (3-ethylbenzothiazoline- 6-sulfonic acid) diammonium salt (ABTS^●+^) [26]. These observations have prompted us to postulate that TMT may serve as a good candidate for the purposes of metal absorption and free radicals scavenging in microorganisms during bio-fuel production.

It is known that the levels of intracellular reactive oxygen species increase in *E. coli* after exposure to *n-*butanol [15]. In this study, we demonstrate that engineered *E. coli* strains expressing OmpC fused MTs could elevate *n-*butanol tolerance by scavenging intra- and extra-cellular free radicals and the fusion protein could still contribute in osmosis via either GB or glucose uptaking.

## Results and discussion

### Alcohols tolerance assay

Alcohol tolerances in a variety of microorganisms have been reported by many previous studies (Table 1). A few naturally occurring microorganisms presented a high alcohol tolerance: as high as 6% n-butanol in *Pseudomonas*[27] and 14% ethanol in *Candida*[3,4]. However, these alcohols are sensitive toxins to *E. coli* as tolerances of n-butanol and ethanol are only 0.5-1% and 4–5%, respectively (Table 1). In this study, we attempted to improve the alcoholic tolerance of *E. coli* via a MTs expression approach.

**Table 1:** Overview of ethanol and n-butanol tolerance in microorganisms

**Microorganism**	**Ethanol tolerance**	**n-Butanol tolerance**	**Reference**
*E. coli*	4 - 5%	0.5 – 1%	[4,28]
	4 - 5%	1.5 – 2%	This work
*Bacillus*	2.5 - 5%	1 – 2.25%	[29,30]
*Clostridia*	4 - 5%	1.2 - 1.5%	[31,32]
*Pseudomonas*	-	6%	[27]
*Lactobacillus*	2.5 - 5%	2.5 - 3%	[4,29]
*Saccharomyces*	9.5 - 11%	1 - 2%	[4]
*Zymomonas*	13%	1 - 2%	[4]
*Pichia*	-	< 1%	[4]
*Candida*	14%	1 - 2%	[4]

**Figure 1:**
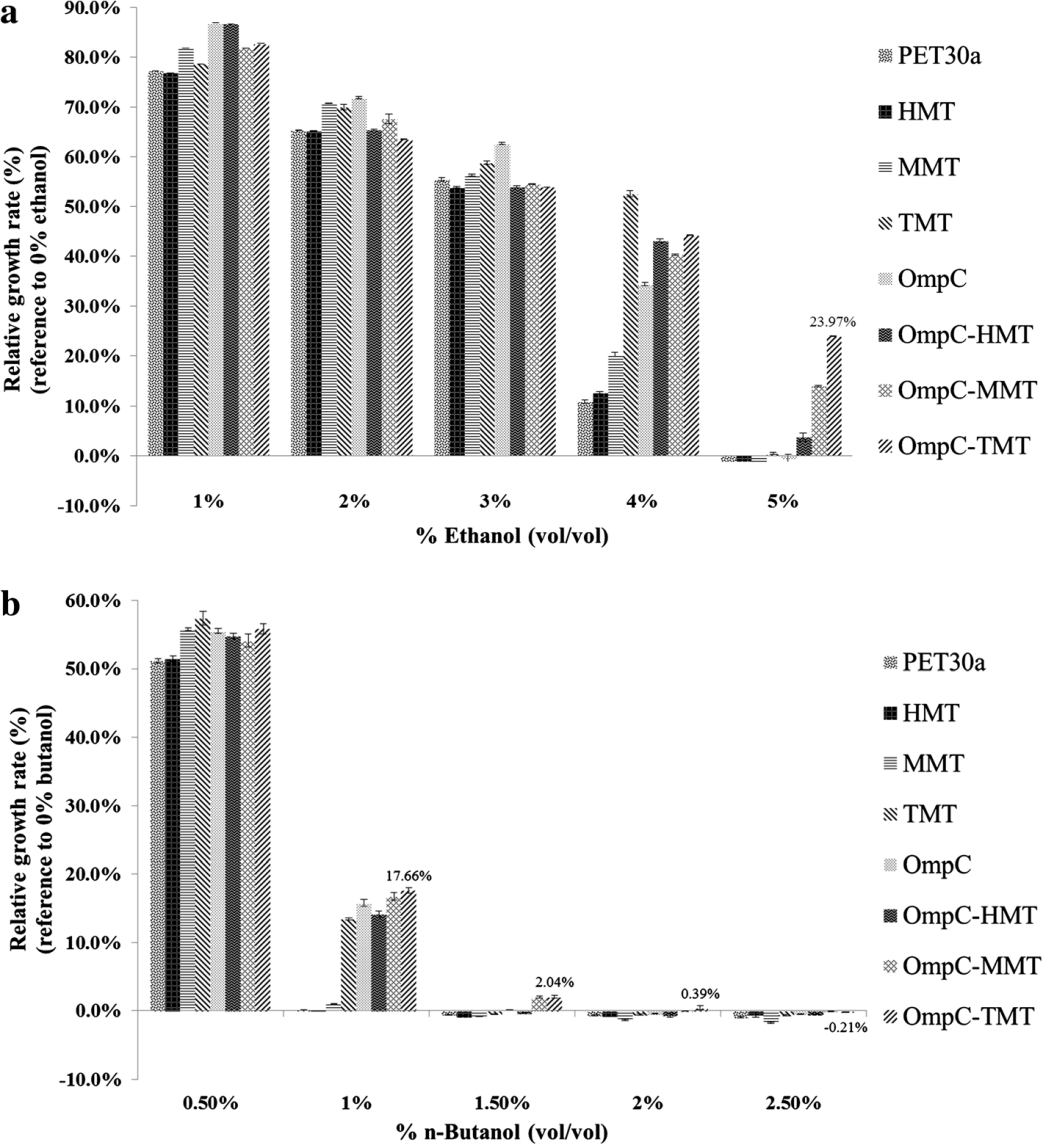
**Alcohol tolerance assay.** The OD_600_ values were measured for engineered *E. coli* strains cultured in PYG medium with different concentrations of **(a.)** ethanol and **(b.)** n-butanol (vol/vol). The relative growth rates were based on the comparison between conditions under alcoholic stresses or not. The values and error bars are based on three replicate experiments.

Therefore, alcohols tolerance measurements for the engineered *E. coli* strains of HMT, MMT and TMT were cytosolic expression, while the OmpC fused MTs strains (OmpC-HMT, OmpC-MMT and OmpC-TMT) were expressed for membrane-targeted MTs (Table 2). The tolerance assays of *n-*butanol and ethanol of these engineered *E. coli* strains were examined from 0% - 2.5% and 0–5%, respectively, and the relative growth rate was defined as the [ (A600) _challenge, *t12*_ − (A600) _callenge, *t0*_ / (A600) _no challenge, *t12*_ − (A600) _no challenge, *t0*_ ] × 100. When either 1–3% ethanol or 0.5% *n-*butanol was added, there was no significant difference among the different engineered *E. coli* strains. When 4% ethanol or 1% *n-*butanol was added, the TMT strain showed the best tolerance among the engineered strains; all subsequent higher concentrations of alcohols yielded no higher tolerance in each of the cytosolic-expressed MT strains (Figure 1). Additionally, we also tested the hypothesis that the strains expressed membrane-targeted MTs that could enhance alcohol tolerance by decreasing damage to the cell membrane. All of the engineered strains expressing MTs on the outer membrane were observed to enhance alcohol tolerance up to 5% ethanol; however, only the OmpC-MMT and OmpC-TMT strains were able to tolerate 1.5% *n-*butanol (Figure 1). Our data indicated that the TMT strains showed a higher capacity of alcohol detoxification than the MMT strains, and the HMT strains showed the lowest detoxification capacity. Furthermore, all of the membrane-targeted MTs strains showed higher alcohol tolerances when compared to strains expressing cytosolic MTs at the higher concentrations of alcohols (1.5% *n-*butanol or 5% ethanol). The membrane-targeted MTs strains showed the better capability of tolerance for both alcohols.

**Table 2:** Strains used in this study

**Strains**	**Genotype and description**	**Reference or source**
*E. coli* BL21	*E. coli* B F^-^ dcm ompT hsdS(rB^-^ mB^-^) gal [malB+]K-12(λS)	Invitrogen
PET30a	*E. coli* BL21/ PET30a , T7 promoter, f1 origin, Km^r^	Novagen, Inc.
HMT	*E. coli* BL21/PET30a;*hmt* (human metallothionein)	[26]
MMT	*E. coli* BL21/PET30a;*mmt* (mouse metallothionein)	[26]
TMT	*E. coli* BL21/PET30a;*tmt* (tilapia metallothionein)	[26]
OmpC	*E. coli* BL21/PET30a;*ompC* (*E. coli* outer membrane protein C)	[26]
OmpC-HMT	*E. coli* BL21/PET30a;*ompC-hmt*	[26]
OmpC-MMT	*E. coli* BL21/PET30a;*ompC-mmt*	[26]
OmpC-TMT	*E. coli* BL21/PET30a;*ompC-tmt*	[26]

In previous studies, MTs were known to increase cellular tolerance to toxins by scavenging free radicals that were produced during stress [33,34]. In this study, it was hypothesized that the increased alcohol tolerance in engineered *E. coli* strains was due to the ability of MTs, particularly the TMT strains, to possess higher scavenging efficiencies as previously reported [26]. Overall, both membrane-targeted MMT and TMT strains were found contributing to 3 times *n-*butanol (0.5% to 1.5%) and 1.25 times ethanol (4% to 5%) greater tolerances, respectively, than the control *E. coli* strains (pET30a). Interestingly, the OmpC over-expressed *E. coli* strains without MTs also enhanced its alcohol tolerance to 1% *n-*butanol and 4% ethanol; this phenomenon was also observed in another study in which an *E. coli* strain EbN1 was observed to tolerate phenol by expressing OmpC [35]. We hypothesize that OmpC might not only act as a membrane-targeted protein but also utilizes its osmoregulative ability, leading to the accumulation of compatible solutes that prevent solvent stress.

### Free radical scavenging ability

**Figure 2:**
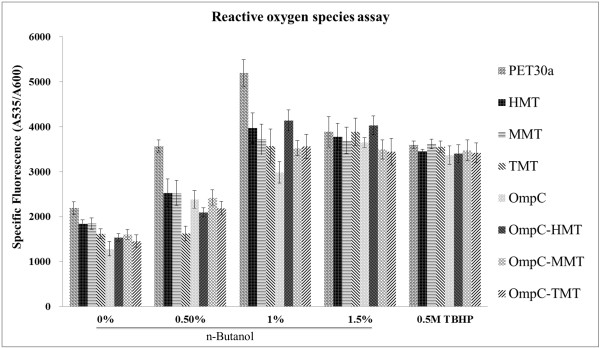
**Quantitative assay of intracellular reactive oxygen species under different n-butanol concentrations.** The OD_600_ values of engineered *E. coli* strains were measured in cells cultured in PYG medium with different concentrations of n-butanol (vol/vol) at 37°C TBHP represents the positive control of the strains cultured with 0% n-butanol, but treated with TBHP (a known stressor that produces intracellular H_2_O_2_). Values are averages of three replicate experiments.

Toxins and stresses are factors of oxidative stress leading to elevated radicals in cells. MTs are well-known antioxidants that scavenge radicals and alcohols are known factors that cause oxidative stress in *E. coli*[35]. It is worthwhile to investigate cytosolic and membrane-targeted MTs, as they function as radical scavengers and increase the host toxin tolerance. In this study, we examined the capacity for MTs to scavenge free radicals when the host cells were treated with 0 to 1.5% n-butanol. We then detected the content of ROS in cells by 5(6)-Carboxy-2',7'- dichlorodihydrofluorescein diacetate (carboxy-H_2_DCFDA) (Figure 2). It was observed that free radicals in all strains increased with increasing concentrations of n-butanol from 0–1.5%. However, both cytosolic- and membrane-targeted expressed MTs strains had lower levels of radicals than the control pET30a strain up to 1% (Figure 2). Moreover, in the lower n-butanol concentrations (less than 1%), the TMT strain showed an increased capacity for scavenging free radicals than either MMT or HMT strains. Notably, the membrane-targeted MTs strains showed elevated radical scavenging capacities than the strains expressing the cytosolic-MTs. In the higher n-butanol (1.5%) treatment, the both membrane-targeted MMT and TMT strains, except OmpC-HMT, showed highest radical scavenging capacities than all of the test strains (Figure 2). These results suggested that the expression of MT proteins could lower the levels of free radicals and enhance the tolerance for n-butanol. Interestingly, non-MT OmpC-only strain also showed the abilities for both lowering the free radicals and enhancement of n-butanol tolerance. In particular, the OmpC strain was observed to have the lowest level of radicals among all of the engineered strains when treated with 0–1% n-butanol (Figure 2). It has been suggested that osmoregulation could enhance solvent tolerance [36] and our results from overexpressing OmpC supported the suggestion. In addition, the slightly increased tolerance capacity of the OmpC-MMT and OmpC-TMT strains under 1.5% *n-*butanol stress (Figure 1b) might be attributed to the combination of both osmosis and elevated extracellular radical scavenging capacities, especially in the presence of increased ROS levels originating from lysed cells. The results of these ROS assays for higher n-butanol concentrations indicate that the slight growth capacities observed in the OmpC-TMT and OmpC-MMT strains (Figure 1) are caused by decreased oxidative stress due to increased scavenging of extracellular radicals.

### The roles of outer membrane (OM) proteins

**Figure 3:**
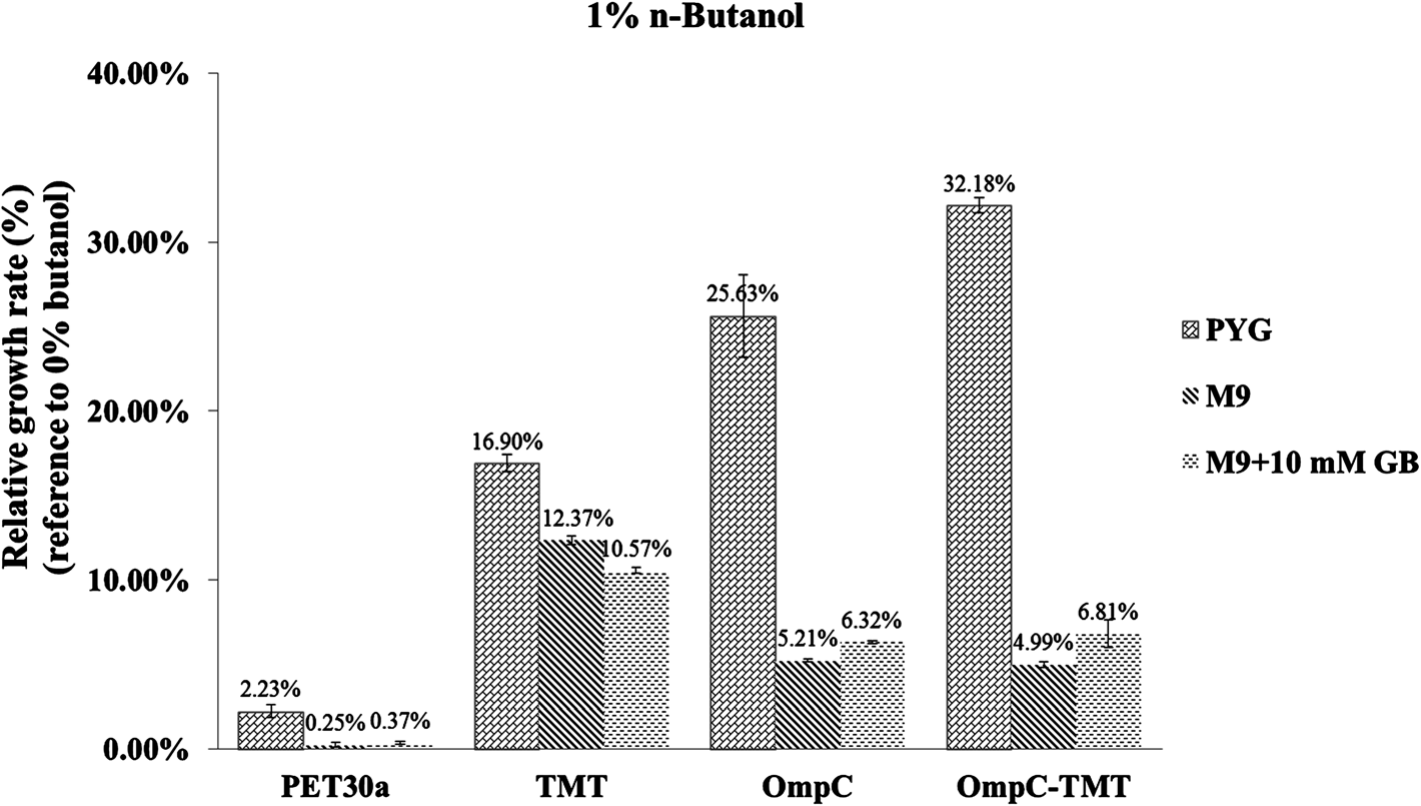
**Assay of osmoregulation capacity in PYG or M9 medium with/without glycine betaine.** The OD_600_ values of engineered *E. coli* strains were measured for cells cultured in PYG or M9 medium with 1% n-butanol (vol/vol) at 37°C. The M9 minimal medium is a simple growth medium containing 1% glucose, sodium chloride and phosphate salt; the compatible solute, GB, was added at 10 mM concentration for evaluation of osmoregulation capacity. The relative growth rates were compared with the strain controls cultured in PYG medium without alcohols.

Previous studies have reported that osmoregulation of a cell can help the uptake of compatible solutes, such as proline, choline, proline betaine and GB, through active transportation by transmembrane proteins such as OmpC in *E. coli*[36,37]. To determine whether n-butanol tolerance is dependent on OmpC presence in our engineered *E. coli* strains, the pET30a, TMT, OmpC and OmpC-TMT strains were cultured in M9 minimal medium containing 1% *n-*butanol and with or without 10 mM GB. After culture for 12 hours at 37°C with 1% n-butanol as stress, the adding of GB in M9 medium could not enhance the growth of TMT stain (even worse). On the other hand, without 10 mM GB, the relative growth rates of OmpC and OmpC-TMT strains were 5.21% and 4.99%, respectively, while tolerances were slightly increased when the same strains cultured with GB (OmpC: 6.32% and OmpC-TMT: 6.81%) (Figure 3). The result indicates that the medium containing GB was not responsible for an increased tolerance capacity for non OmpC overexpressing strain but for those OmpC related strains GB could contribute to their tolerance (Figure 3). From these results, we suggested that strains overexpressing OmpC were accumulating compatible solute through OmpC into cytosol and lead to slightly elevating n-butanol tolerance. It is also suggested that our construction strategy of OmpC-MT fusion protein for membrane targeting did not abolish the function of OmpC, as the dual functional OmpC-MT fusion protein could not only regulate compatible solutes but also reduce radicals to elevate the host’s n-butanol tolerance.

In PYG medium, it was found that the growth rate of the OmpC overexpressed strains were nearly four times faster than other strains without overexpressed OmpC protein (Figure 3). Meanwhile, the OmpC overexpressed strains cultured in M9 minimal medium showed that growth rates were nearly 5 to 6.5 times lower than the same strains cultured in PYG medium. It was also observed that the growth rate of the TMT strain in M9 medium was 1.65 times lower when compared to the rate observed in PYG medium cultured TMT strain. Previous reports have observed that the porins OmpF and OmpC are differentially regulated by glucose concentrations because the two porins constitute the main glucose entry channels into the periplasm when the carbon source is present at a higher concentration of 0.2 mM (0.036 g/l) [38]. Cellular growth rate has been correlated to the uptake of glucose via OmpF and OmpC. Based on these evidences, it suggested that overexpressed OmpC could not only increase growth through osmoregulation of compatible solutes such as GB but also regulating glucose-uptake-capacity in M9 minimal (2 g/l glucose) and PYG (10 g/l glucose) medium, It is also found that the cytosolic TMT strain showed higher growth rates than that of the OmpC and OmpC-TMT strains in M9 minimal medium. As non-rich medium could generate radicals in cytoplasmic matrix, this phenomenon might be mostly related to the free radicals scavenging ability of cytosolic TMT.

### Tolerance assay of lignocellulose pretreatment’s toxins

In the bio-fuel industry, the pretreatment of lignocellulose substrate is a complex process requiring dilute acid and steam pretreatment and involving many toxins, including furfural, hydroxymethylfurfural and heavy metals [39]. In this study, the engineered *E. coli* strains were also used to test the toxin tolerance of these compounds.

**Figure 4:**
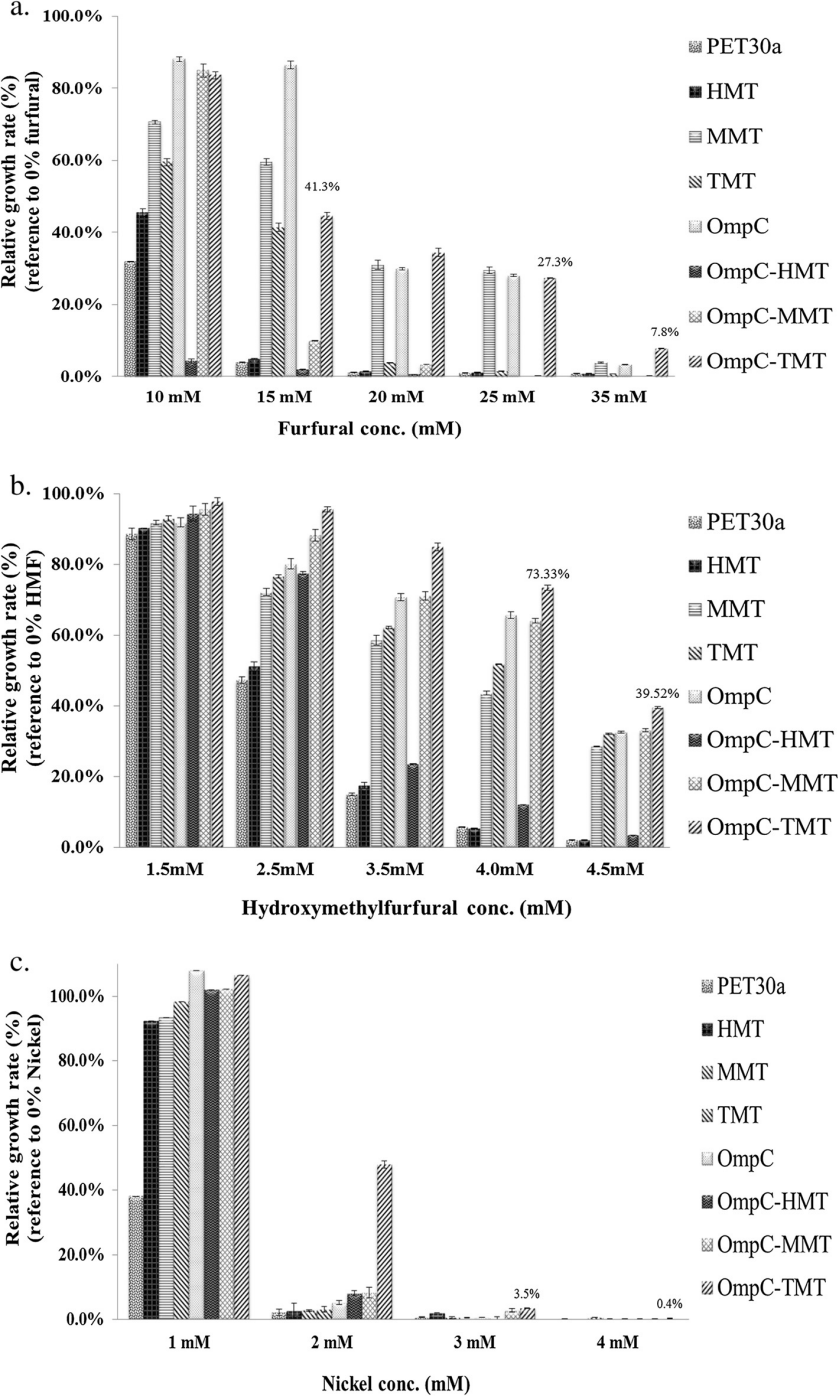
**Tolerance assay of lignocellulose pretreatment’s toxins.** Toxin tolerance assays were conducted with different concentrations of **(a.)** furfural, **(b.)** hydroxymethylfurfural, and **(c.)** nickel. The OD_600_ values of engineered *E. coli* strains cultured in PYG medium were also measured. The relative growth rates were compared with the strain controls cultured in PYG medium without toxins.

Furfural and hydroxymethylfurfural (HMF) are dehydration products of hemicellulose hydrolysates and can be used as fermentation inhibitors but are also potential toxins [39-41]. The data of relative growth in furfural-positive media of the engineered *E. coli* strains indicated that all but not the both HMT-expressed strains could raise the furfural tolerance capacity in 15 mM (Figure 4a). The MMT strain, which expressed the cytosolic MTs from mouse, showed best growth among cytoslically expressed MTs strains in all furfural concentrations. The OmpC-TMT strain, which expressed the membrane-targeted MTs from tilapia, showed the highest furfural tolerance capacity (35 mM). However, the TMT strain, which expressed cytosolic MTs from tilapia, did not show a tolerance enhancement from 20 mM furfural, relative to the MMT strain. In the HMF stress tests, both cytosolic-expressed and membrane-targeted MTs from tilapia and mouse showed a high relative growth rate (30 to 40%) in 4.5 mM HMF (Figure 4b). The OmpC strain, which only overexpressed OmpC protein, also increased toxin tolerances to both furfural and HMF. This could also be explained by its osmoregulation ability. Interestingly, the tolerances of MMT performed better than TMT in furfural (Figure 4a), Our previous studies [26], we found MT display different scavenging capacity between two kinds of radicals (ABTS^●+^ and DPPH^●^). We suggested that MMT prefers to scavenge the type of ROS generated from furfural.

Furthermore, it is expected that the OmpC-MTs fusion strategy would show increased growth rates due to the combination of osmoregulation and MTs extracellular free radical scavenging abilities. Indeed, the OmpC-TMT strain was observed to have twice and 1.3 times of the growth rate compared to the OmpC strain in furfural and HMF, respectively, and better than pET30a strain (Figure 4a and 4b).

Heavy metals, such as nickel, may also be present in the host cell environment and are likely sourced from the substrates or its solubilized byproducts during lignocellulose pretreatment [39]. In addition to the other toxins, nickel tolerance was also tested. All engineered *E. coli* strains showed a significant nickel tolerance compared to the control strain (pET30a strain) in 1 mM nickel (Figure 4c). When 2 mM nickel-supplemented media was used, only the OmpC-TMT strain showed a distinguished relative growth over the control strain (47.9%). It is predicted that the *E. coli* strains expressing the OmpC-TMT protein could chelate metals in the external milieu and could also decrease the toxin-induced oxidative stress in the cytosol. This mechanism was also suggested by a previous study, which used Hg to test toxin tolerance in *E. coli*[26].

## Conclusions

This study uses a novel approach to develop *E. coli* strains that expresses cytosolic and membrane-targeted MTs to improve cell tolerance capacity of toxins derived from fermentation process. From results, we suggested that our construction strategy of OmpC-MT fusion protein for membrane targeting did not abolish the function of OmpC, as the dual functional OmpC-MT fusion protein could not only regulate compatible solutes and glucose but also reduce radicals to elevate the host’s toxins’ tolerances.

## Materials and methods

### Reagents

All of the chemicals and reagents used were purchased from the Sigma-Aldrich Co. USA, unless mentioned otherwise. The reagents, when available, were molecular biology grade. All solutions were prepared using these reagents and sterile distilled water.

### Bacterial strains, culture media and culturing conditions

MTs expressing engineered constructs, protein expression and their locations in recombinants *E. coli* hosts were confirmed in our previous study [26]. Batch cultures were grown in 10 ml PYG medium (5 g of peptone, 10 g of yeast extract, 10 g of glucose, 5 g of tryptone, 40 mg of K_2_HPO_4_, 19.2 mg of MgSO_4_.7 (H_2_O), 8 mg of CaCl_2_, 40 mg of KH_2_PO_4_, 0.4 g of NaHCO_3_, 80 mg of NaCl and 1.1 mg of FeSO_4_.7 H_2_O) or M9 (AMRESCO-J863) media. Each engineered *E. coli* strain, including PET30a, HMT, MMT, TMT, OmpC, OmpC-HMT, OmpC-MMT and OmpC-TMT (Table 2), were grown in medium supplemented with 30 μg/mL kanamycin at 37°C. When culture density reached O.D. 0.6, isopropyl-β-D-thiogalactopyranoside (IPTG) was added to for a final culture concentration of 0.6 mM. After eight hours of incubation, cells were harvested for tolerance experiments. All solvent concentrations in media are reported as% (v/v).

### Tolerance assay of toxins

The above described (Table 2) pre-cultures of *E. coli* BL21 (DE3) strains, including different pET-30a plasmids, were inoculated at an initial O.D. of 0.1 in PYG medium containing 0.6 mM IPTG and 0–2.5% of n-butanol (v/v) or other toxins (furfural, hydromethylfurfural (HMF) and nickel). The cells were assessed after 12 hours of growth at 37°C. The relative growth rates were presented as the cell densities measured at a wavelength of 600 nm by spectrophotometer (GE Healthcare Life Sciences "GeneQuant 1300). Densities of toxin-treated cultures were normalized by the density of their respective toxin-free controls under otherwise same growth conditions [42]. From each tolerance assay, percent tolerance relative to unchallenged cultures was estimated at each challenge level and sample time as follows:

$$\begin{array}{l} \%\mathrm{tolerance}=\frac{\left(A600\right)\;\mathrm{challenge},t12-\left(A600\right)\;\mathrm{challenge},t0}{\left(A600\right)\;\mathrm{no}\;\mathrm{challenge},t12-\left(A600\right)\;\mathrm{no}\;\mathrm{challenge},t0}\\ \;\;\;\;\times 100 \end{array}$$

### Reactive oxygen species detected by carboxy-H_2_DCFDA under n-butanol stress

The engineered *E. coli* strains were pre-cultured in PYG medium containing 0%, 0.5%, 1%, 1.5%, 2% and 2.5% *n-*butanol. Aliquots of 100 μl of pre-cultured strains were each re-suspended in 5 ml M9 medium; 140 μl of each diluted sample was transferred to a 96-well plate followed by incubation at 37°C for 45 min. The assay method was adapted from a previous study [15]. All samples were treated with 10 μl of 25 mM carboxy-H_2_DCFDA (Invitrogen, Co., Carlsbad, CA) and incubated at 37°C for 15 min. The optical density at 600 nm and the fluorescence excitation/emission at 535/600 nm of each sample were measured by a plate reader. Tert-butyl hydroperoxide (TBHP) (Invitrogen, Carlsbad, CA) is a known stressor that produces intracellular H_2_O_2_; a set of positive controls for the ROS assay were prepared with the strains cultured without n-butanol and treated by same steps as above except with an initial 45 min incubation of 10 μl of 7.78 M TBHP.

## Abbreviations

ROS: Reactive oxygen species; PYG: Medium containing peptone, yeast extract and glucose; OM: Outer membrane; HMF: Hydroxymethylfurfural; GB: Glycine betaine.

## Competing interests

The authors declare no competing interests.

## Authors’ contributions

Chin, W. C. performed the experiments, analyzed the data and drafted the manuscript; Lin, K. H. helped in some experimental work. Lin, K. H. and Chang, J. J. contributed in data interpretation; Chin, W. C. and Lin, K. H. wrote and revised the manuscript; Chin, W. C. and Huang, C. C. designed the study; Huang, C. C. coordinated the study. All authors read and approved the final manuscript.
